# Supplementary material for: Rheological Analysis and Evaluation of Measurement Techniques for Curing Poly(Methyl Methacrylate) Bone Cement in Vertebroplasty
Source: ACS Biomater Sci Eng. 2024 Jun 5;10(7):4575–86. doi: 10.1021/acsbiomaterials.4c00417 (PMC11235098; doi:10.1021/acsbiomaterials.4c00417)
Supplement: Supplementary file 1 — ab4c00417_si_001.pdf [file ab4c00417_si_001.pdf]

Rheological Analysis and Evaluation of Measurement Techniques for the Curing Polymethylmethacrylate Bone Cement in Vertebroplasty - Supporting Information

Zubin Trivedi,[*,†,‡,⊥] Jacek K. Wychowaniec,[¶,⊥] Dominic Gehweiler,[¶] Christoph M. Sprecher,[¶] Andreas Boger,[§] Boyko Gueorguiev,[¶] Matteo D'Este,[¶] Tim Ricken,[‡] and Oliver Röhrle[†,∥]

†*Institute for Modelling and Simulation of Biomechanical Systems, University of Stuttgart, Pfaffenwaldring 5a, 70569 Stuttgart, Germany*

‡*Institute of Structural Mechanics and Dynamics in Aerospace Engineering, University of Stuttgart, Pfaffenwaldring 27, 70569 Stuttgart, Germany*

¶*AO Research Institute Davos, Clavadelerstrasse 8, 7270 Davos, Switzerland*

§*Ansbach University of Applied Sciences, Residenzstraße 8, 91522 Ansbach, Germany*

∥*Stuttgart Center for Simulation Science (SC SimTech), Pfaffenwaldring 5a, 70569 Stuttgart, Germany*

⊥*These authors contributed equally to this work*

E-mail: zubin.trivedi@imsb.uni-stuttgart.de

The data presented in the study and the rheometer settings for the tests can be found in the data repository under the link https://doi.org/10.18419/darus-4004.

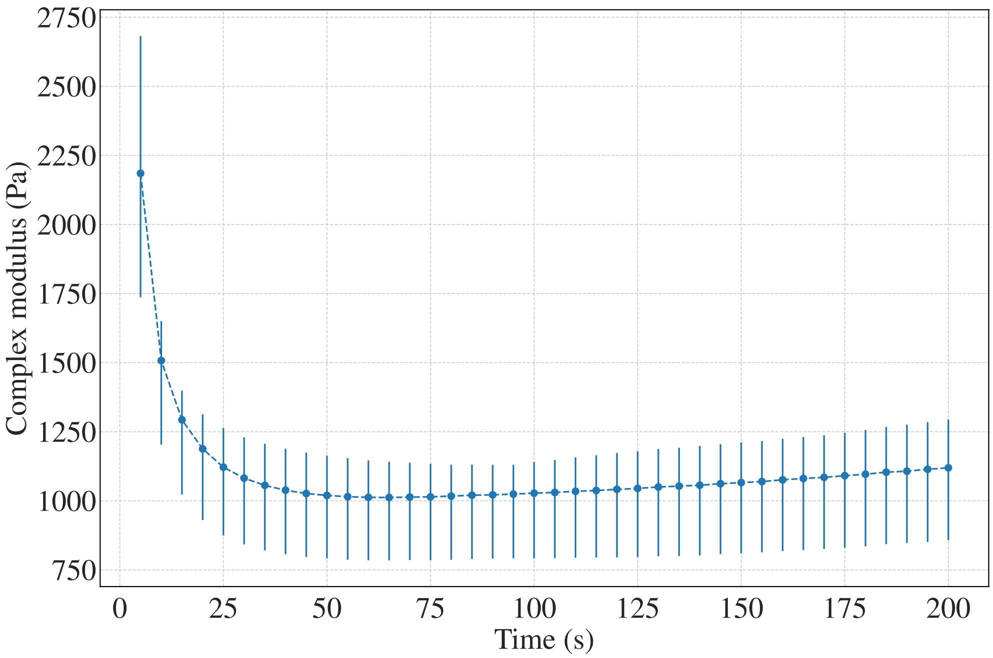

Figure S1: Results of benchmark test on the bone cement prepared by our mixing process. The benchmark test was employed to check the repeatability of the bone cement behaviour from the mixing process. In the test, the bone cement sample was subjected to oscillations at a maximum torque of 3 mNm and 1 Hz frequency at 23 °C, with a measuring point every 5 seconds. The mixing method produced qualitatively reproducible results with quantitative deviations of up to 25%.

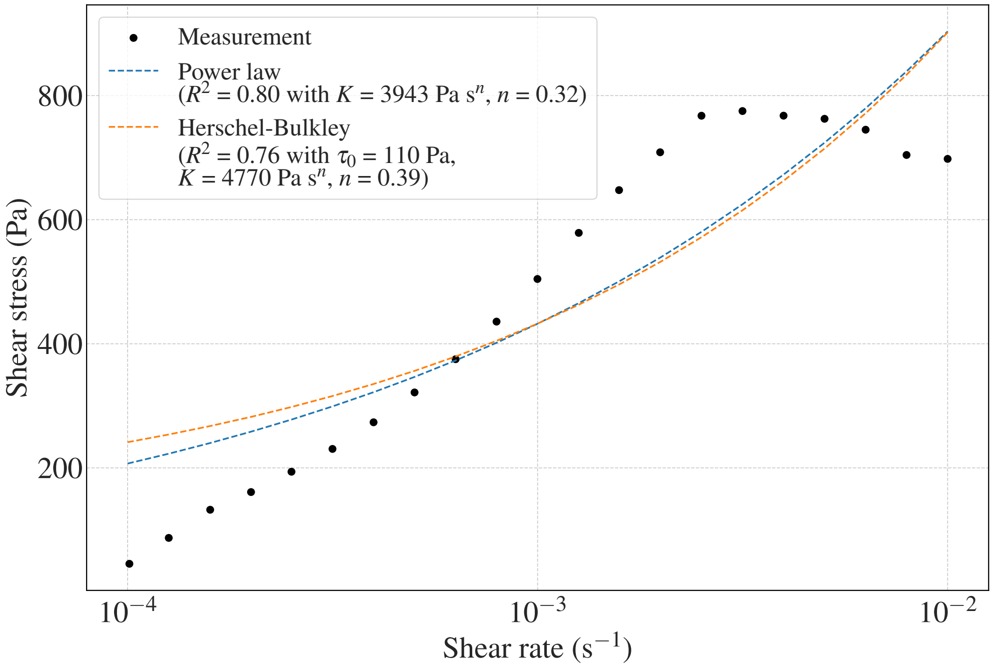

Figure S2: Comparison of fitting using the Herschel-Bulkley and the power law rheological models on the rotational shear rate sweep test from 10^{-4} to 10^{-2} s^{-1} (Test Rh7a-3)

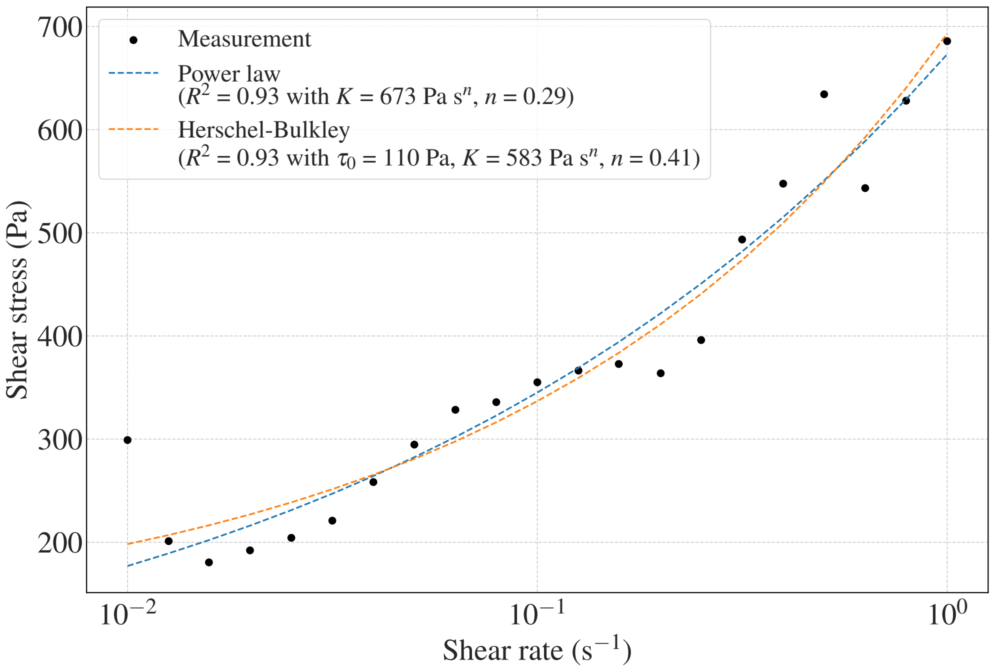

Figure S3: Comparison of fitting using the Herschel-Bulkley and the power law rheological models on the rotational shear rate sweep test from 10^{-2} to 1 s^{-1} (Test Rh7a-2)

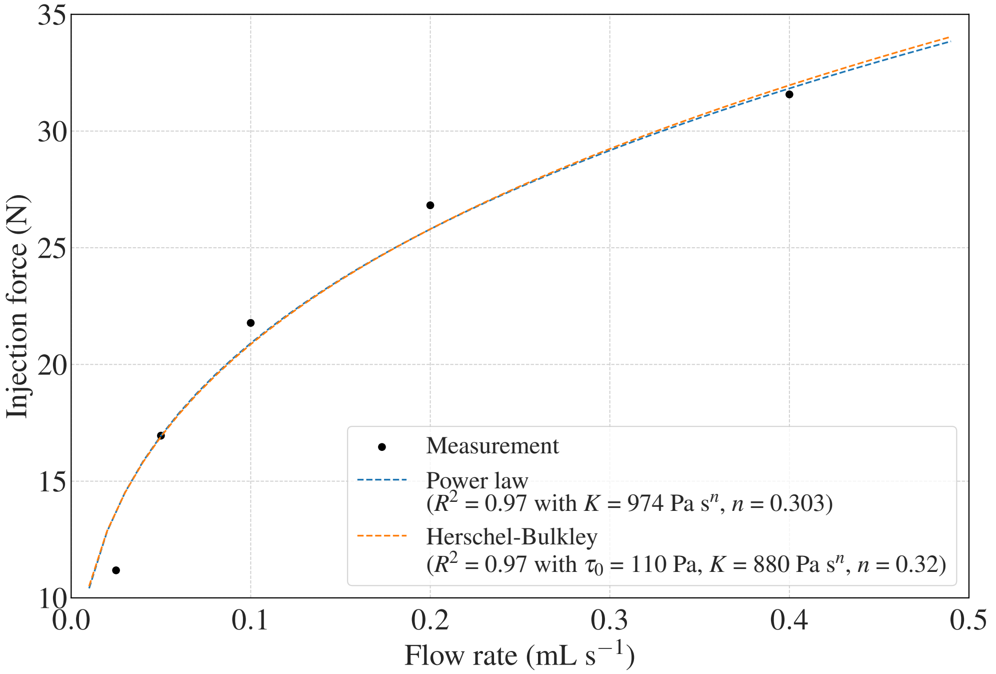

Figure S4: Comparison of fitting using the Herschel-Bulkley and the power law rheological models on the injection force measurement tests at various flow rates (Tests Inj1–Inj5)
